# Assessing anesthesiology residents’ out-of-the-operating-room (OOOR) emergent airway management

**DOI:** 10.1186/s12871-017-0387-2

**Published:** 2017-07-15

**Authors:** Lauryn R. Rochlen, Michelle Housey, Ian Gannon, Shannon Mitchell, Deborah M. Rooney, Alan R. Tait, Milo Engoren

**Affiliations:** 1 Department of Anesthesiology, University of Michigan, 1500 E. Medical Center Drive, 1H247 University Hospital, SPC 5048, Ann Arbor, MI 48103 USA; 2 Department of Anesthesiology, University of Michigan, 2800 Plymouth Rd, NCRC, Bldg 16 G149S, Ann Arbor, MI 48109 USA; 3 Department of Learning Health Sciences, University of Michigan, G2400 Towsley Center, 1500 E. Medical Center Drive, Ann Arbor, MI 48109-5201 USA

**Keywords:** Urgent airway management, Outside the OR intubations

## Abstract

**Background:**

At many academic institutions, anesthesiology residents are responsible for managing emergent intubations outside of the operating room (OOOR), with complications estimated to be as high as 39%. In order to create an OOOR training curriculum, we evaluated residents’ familiarity with the content and correct adherence to the American Society of Anesthesiologists’ Difficult Airway Algorithm (ASA DAA).

**Methods:**

Residents completed a pre-simulation multiple-choice survey measuring their understanding and use of the DAA. Residents then managed an emergent, difficult OOOR intubation in the simulation center, where two trained reviewers assessed performance using checklists. Post-simulation, the residents completed a survey rating their behaviors during the simulation. The primary outcome was comprehension and adherence to the DAA as assessed by survey responses and behavior in the simulation.

**Results:**

Sixty-three residents completed both surveys and the simulation. Post-survey responses indicated a shift toward decreased self-perceived familiarity with the DAA content compared to pre-survey responses. During the simulation, 22 (35%) residents were unsuccessful with intubation. Of these, 46% placed an LMA and 46% prepared for cricothyroidotomy. Nineteen residents did not attempt intubation. Of these, only 31% considered LMA placement, and 26% initiated cricothyroidotomy.

**Conclusions:**

Many anesthesiology residency training programs permit resident autonomy in managing emergent intubations OOOR. Residents self-reported familiarity with the content of and adherence to the DAA was higher than that observed during the simulation. Curriculum focused on comprehension of the DAA, as well as improving communication with higher-level physicians and specialists, may improve outcomes during OOORs.

**Electronic supplementary material:**

The online version of this article (doi:10.1186/s12871-017-0387-2) contains supplementary material, which is available to authorized users.

## Background

Emergent airway management outside of the operating room (OOOR) has been shown to be associated with an estimated complication rate of 10–39% and an increased incidence of difficult or failed intubation. [1–5] Complications include hemodynamic instability, oxygen desaturation, aspiration, esophageal intubation, multiple laryngoscopy attempts, cricothyroidotomy, and death. [2, 6–8] Mortality rates following emergent intubation have been observed to be as high as 46%. [3] Interventions intended to improve outcomes following emergent intubation have included the development of novel training programs, a pre-procedure checklist and a care bundle to ensure appropriate evaluation and a post-event debriefing. [1, 9, 10].

At academic institutions, anesthesiology residents may be responsible for airway management outside of the operating room, either independently or with another resident. Poor judgment in conjunction with lack of education and training may be the leading preventable causes of adverse outcomes related to airway management. [11, 12] However, a survey performed by Hagberg et al. revealed that, at most, only 33% of anesthesiology residency training programs offer focused instruction on difficult airway management. [13] For those programs that do offer such courses, the educational methods are largely traditional, and include didactic lectures and videos. A more recent review by Pott et al. revealed that simulation and mannequin-based education are increasingly being integrated into anesthesiology resident airway management training. [14].

The role of simulation-based curriculums for training in airway management has been examined with varying results. [15–19] Following a dedicated simulation-based curriculum in management of the difficult airway, Borges et al. found incomplete adherence to the American Society of Anesthesiologists’ (ASA) Difficult Airway Algorithm (DAA) by staff anesthesiologists. [15, 20] On the other hand, Kuduvalli et al. showed improvement and skill retention in simulated difficult airway management at 6–8 weeks following training. [17] More recent studies have examined the effect of training in a DAA and cricothyrotomy technique prior to simulation testing. [21, 22] These studies have demonstrated improved compliance with algorithms and technical performance of cricothyrotomy in anesthesiology residents. However, none of these studies included independent difficult airway management by residents *outside of the operating room*.

By comparing questionnaire responses with actions performed in a simulated scenario, we evaluated resident familiarity with content and adherence to the ASA DAA in regards to emergent airway management OOOR. Furthermore, we assessed the residents’ reasoning and frequency of requesting anesthesiology faculty and otolaryngology presence during these events. Finally, we examined differences in behavior based upon resident experience.

## Methods

This protocol was determined to be exempt from full Institutional Review Board review as the research was conducted in an established educational program using accepted educational strategies.

Ninety anesthesiology residents covering post-graduate years 2–4 from the University of Michigan Department of Anesthesiology were eligible to participate. Exclusion criteria included resident refusal or inability to schedule simulation center testing within the allotted time. Participating residents were guaranteed that their performance in this study would not affect their evaluations within the anesthesiology department.

### Data collection

#### Pre-simulation survey 

A survey was developed to ascertain residents’ perceptions of their OOOR urgent airway management (Additional file 1: Appendix 1). The survey consisted of open-ended and multiple choice questions based on information from current literature regarding airway management [20, 23, 24]. The survey was reviewed for content validity by 5 faculty from the University of Michigan Department of Anesthesiology with expertise in simulation or board-certification in Critical Care Medicine. The survey also captured perceived need and perceived benefit of dedicated teaching curriculum for OOOR emergent airway management. The survey was administered using Qualtrics Survey Software; a link to the survey was emailed to all residents one month prior to the simulation experience. Residents were asked to give their pager number in order to link their survey results to their performance in the simulator. The data received from the survey were only shared with those involved with the project.

#### Simulation scenario

Following completion of the survey, each resident was asked to perform emergent airway management OOOR in the simulation center. The objectives were to assess residents’ performance during a difficult airway scenario, including adherence to the DAA and to determine which tasks are performed most frequently and which are omitted or ignored. Each resident only went through the simulation once, with one possible intubation.

Scenario development was based on prior “Cannot intubate/cannot ventilate” scenarios published in the anesthesiology simulation literature. [17, 25, 26] The scenario described a situation wherein the resident had just transferred a post-operative patient to the trauma-burn intensive care unit (ICU), and was now being asked by an ICU nurse to evaluate a different patient in the unit. The patient was described as a 25 year old male who was involved in a motorcycle accident earlier that day. The nurse is concerned about acute respiratory failure. The resident was informed that the ICU and anesthesiology airway teams are unavailable, as they are responding to a cardiac arrest in the emergency room. Using SimMan® (Laerdal Medical, Stavanger, Norway) software, vital signs and airway anatomy were pre-programmed, and were the same for each resident testing session. To enhance the fidelity of the scenario, the difficult airway settings were pre-programmed and a hard cervical collar was placed. Complete details of the scenario are attached in Additional file 2: Appendix 2. In order for airway management equipment and medications to be present, residents were required to ask the nurse confederate in the scenario to bring them into the room. Residents could also specifically request that a GlideScope be brought into the room.

Simulation performance was evaluated using a checklist of tasks determined to be important by the anesthesiology faculty with at least 5 years of clinical and simulation experience and adapted from previous studies [9, 10, 17, 27, 28] (Additional file 3: Appendix 3). The checklist categories were: airway evaluation, patient preparation, equipment preparation, and airway management. Tasks were rated as complete, incomplete or not applicable. Two of three raters (LRR, SM, EP), experienced with simulation and assessment, independently evaluated each resident’s performance in real-time.

#### Post-simulation survey

Residents were asked to complete a post-simulation survey immediately following the completion of their simulation. The post-simulation survey included questions from the pre-survey to evaluate changes from pre- to post-testing. Residents were also asked to evaluate the fidelity of the scenario and their prior experience with cricothyrotomy (Additional file 4: Appendix 4). Development and content validity testing were the same as for the pre-simulation survey. Administration of the post-survey was also through Qualtrics.

### Statistical analysis

Inter-rater reliability was assessed using the weighted kappa coefficient for the two rater pairs. The primary outcome was resident familiarity and adherence to the ASA DAA as assessed by survey responses and behaviors observed during the simulation. Resident responses to the surveys were linked to data from the simulation. If initial laryngoscopy attempt was unsuccessful, or no laryngoscopy attempt was made, accurate adherence to the DAA was defined as completion of any of the following steps, regardless of order: second attempt with a different laryngoscopy blade, placement of laryngeal mask airway (LMA), placement of alternative airway device, or consideration of cricothryoidotomy. For analyses, residents were grouped according to whether they were ultimately successful, unsuccessful or never attempted an intubation; these groups are denoted as S, U and NA, respectively. Questionnaire responses and simulation behaviors were described with frequencies and percentages. Secondary outcomes included residents’ self-reported frequency of requesting faculty assistance and their behavior in the simulation and whether differences exist in resident behavior based upon year of training (junior versus senior resident). Junior residents were defined as clinical anesthesia year 1 or 2 (CA1, CA2), and senior residents were clinical anesthesia year 3 (CA3) based on our institutional policy that only CA3 residents are permitted to intubate during OOOR airway management events. Chi-square and Fisher’s exact tests were used to compare differences in simulation behavior based upon year of training. *P* < 0.05 denoted statistical significance. All analyses were done using SPSS 21.0 software (SPSS Inc., Chicago, IL).

## Results

Sixty-three residents (26 CA1, 19 CA2, and 18 CA3) completed both pre- and post-surveys and the simulation. Twelve additional residents completed the pre-simulation survey but were not able to be scheduled for the simulation and were therefore excluded from analysis. Residents were not required to answer all questions on the surveys; therefore, denominators differed across questions. One resident refused participation and 26 were unable to attend the simulation during the allotted time. Inter-rater reliability estimates across all checklist items were *κ* = 0.67 and *κ* = 0.62 for the two rater pairs. Based upon post-survey responses, most residents (54/63; 86%) agreed that the simulated scenario was realistic.

### Resident adherence to and familiarity with ASA DAA content

**Fig. 1:**
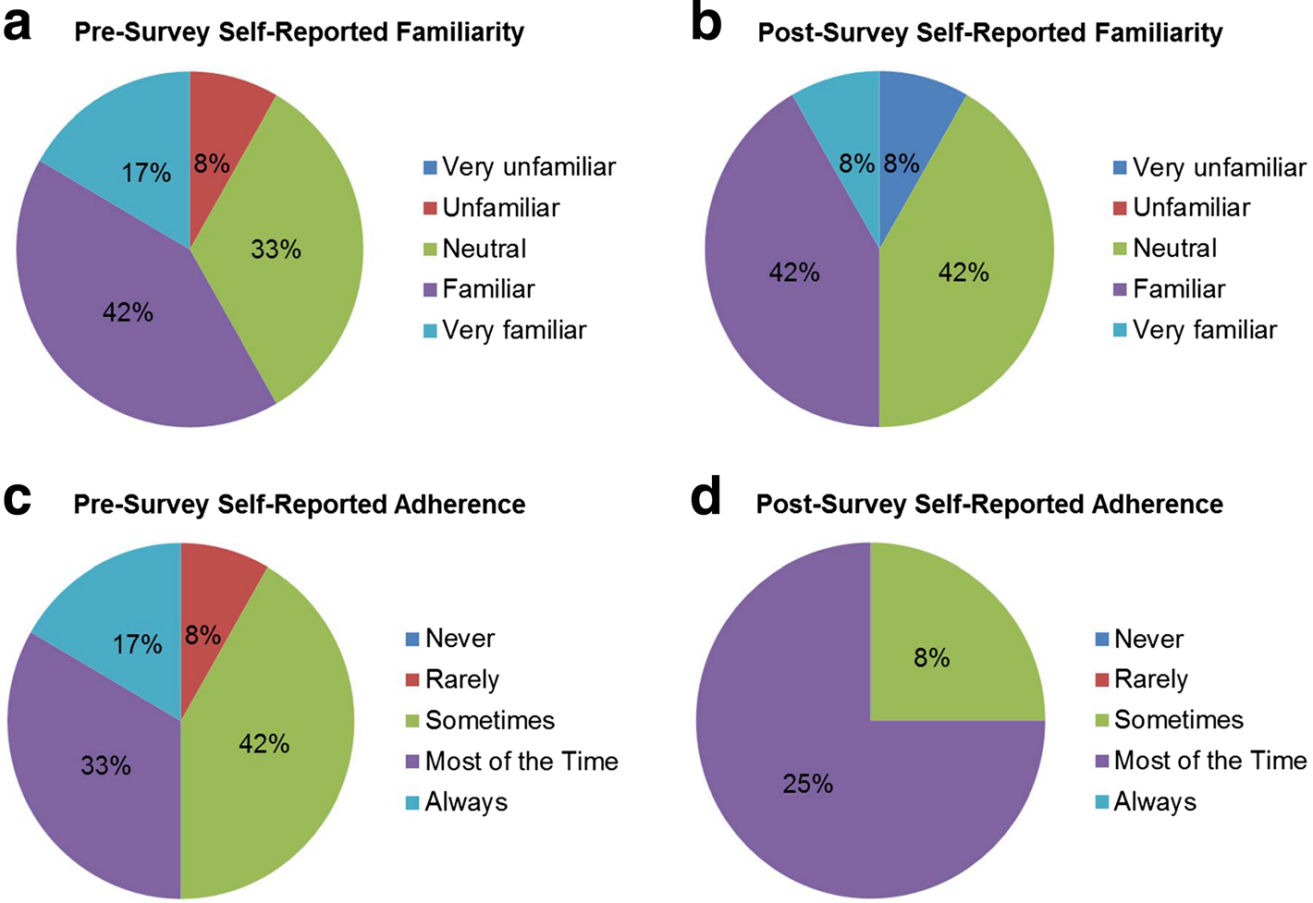
**a**-**d** Reported familiarity with the content of and adherence to the DAA for those residents who did not correctly adhere to the DAA during the simulation

Based on pre-survey responses, 75% (47/63) of residents reported being at least familiar with the content of the ASA DAA and 80% (49/61) of residents reported adhering to the ASA DAA most of time during OOOR intubations. Overall, post-survey responses indicated a shift toward decreased familiarity with the content of the DAA; 68% (43/63) (*p* = 0.15) of residents reported familiarity on the post-test. Fifty-one of 63 (81%) residents appropriately adhered to the DAA during the simulation. Reported familiarity and adherence for those residents not adhering to the DAA are shown in Fig. 1. Twenty-eight of 63 (44%) had previously attended a difficult airway course sponsored by our department. Of those 28 residents, 25 adhered to the DAA during the simulation. Of the 35 residents who had not attended the course, 26 adhered to the DAA during the simulation. (*p* = 0.13).

In the simulation, 22 of the 63 total residents achieved successful intubation (35%), 22 were unsuccessful (35%) and 19 did not attempt an intubation (30%).

Of the 22 residents who were successful at intubation (S), 17 (77%) were successful on their initial laryngoscopy attempt. Two residents were successful on a second attempt with the same blade using an intubating stylet (bougie), and 3 were successful on a second attempt using a different blade. The 5 residents who were not successful on their first attempt were considered to have adhered to the DAA as they completed an appropriate subsequent attempt.

Of the 22 residents, who were unsuccessful with intubation (U), 68% followed the DAA appropriately (i.e., completed any appropriate subsequent step). Despite an initial unsuccessful attempt, 3 (14%) residents made a second attempt with a different blade, 2 (9%) residents considered an alternative device, 10 (46%) placed a laryngeal mask airway (LMA) and 10 (46%) prepared for cricothyroidotomy.

Of the 19 residents who did not attempt an intubation (NA), 74% followed the DAA appropriately. The majority (17; 89%) attempted initial bag mask ventilation; only 6 residents (31%) considered LMA placement, and 5 residents (26%) initiated cricothyroidotomy.

### Requesting anesthesiology faculty and otolaryngology (ENT) presence

Based on pre-survey responses, 22 (32%) of residents reported that they request faculty “sometimes” or “very often”, and 7 (11%) of residents reported that they request ENT presence “sometimes” at OOOR urgent intubations. During the simulation, 25% of residents requested anesthesiology faculty and 1% requested ENT. Only 5 residents requested both faculty and ENT.

The top three reasons for requesting faculty were “history of difficult airway” (44/61, 72%), “concern for difficult airway” (11/61, 18%), and “patient instability” (6/61, 10%); two residents did not complete this question. The top three reasons for requesting ENT presence were “potential for cricothyroidotomy” (40/57, 70%), “history of difficult airway” (13/57, 23%) and “concern for difficult airway” (4/57, 7%); 6 residents did not respond to this question.

Of the 22 residents who were unsuccessful with intubation during the simulation, 6 (27%) requested faculty, 7 (31%) requested ENT, and 9 (41%) requested neither. Of the 19 residents, who did not attempt intubation, 4 (21%) requested faculty presence, 5 (26%) requested ENT, and 10 (53%) requested neither.

### Difference between senior and junior residents

In total, 18 senior residents and 45 junior residents completed both surveys and the simulation. Based on pre-survey responses, senior residents were more familiar with the content of the DAA compared to junior residents (95% vs. 67%, respectively, *p* = 0.026). As seen in Table 1, there were no significant differences between senior and junior residents’ ability to successfully intubate (*p* = 0.316). In the unsuccessful intubation group, senior residents were more likely to attempt a second laryngoscopy with a different blade compared to junior residents (*p*-value = 0.005).

**Table 1:** Frequencies and percentages for DAA behaviors during the simulation by residency class

	Junior Resident N (%)	Senior Resident N (%)	*p*-value
Total	*n* = 45	*n* = 18	
Successful Intubation			
Yes	14 (31)	8 (44)	
No	31 (69)	10 (56)	0.316
Attempts Bag-Mask Ventilation (BMV)			
Yes	40 (70)	17 (83)	
No	5 (30)	1 (17)	0.664
First Attempt Direct Laryngoscope (DL)			
Yes	21 (34)	9 (53)	
No	21 (34)	8 (47)	
N/A^a^	3 (5)	0 (0)	0.540
First Attempt GlideScope			
Yes	12 (27)	7 (39)	
No	24 (53)	8 (44)	
N/A^a^	9 (20)	3 (17)	0.634
Unsuccessful Intubation Group	*n* = 31	*n* = 10	
Unsuccessful Intubation: Second Attempt with Different Blade			
Yes	0 (0)	3 (30)	
No	13 (42)	4 (40)	
N/A^a^	18 (58)	3 (30)	0.005
Unsuccessful Intubation: Attempts Laryngeal Mask Airway (LMA) Placement			
Yes	12 (39)	4 (40)	
No	19 (61)	6 (60)	1.000
Unsuccessful Intubation: Attempts Cricothyroidotomy			
Yes	10 (32)	5 (50)	
No	21 (68)	5 (50)	0.311

^a^N/A denotes that a resident did not attempt that portion of the checklist

## Discussion

Competence in airway management is arguably the most important skill an anesthesiologist should possess. Yet serious adverse events such as death and hypoxemic neurologic injury related to difficulty with intubation remain common. [17, 29] With only approximately one-third of anesthesiology residency programs offering focused training in advanced airway management, anesthesiology residents may not have sufficient clinical exposure to be considered experts in difficult airway management. [30] Although there has been evidence that faculty presence at these events decreases the incidence of complications, this may not be feasible at all institutions due to lack of available resources. [3].

Previous research analyzing complications of airway management revealed that patient characteristics, lack of education/training, poor judgment, equipment/resource failure and poor communication were the most common factors which contributing to adverse events. [8] Educational practices that increase expertise and define superior performance have included deliberate practice, immediate expert feedback, problem-solving and evaluation with the opportunity to repeat performance and modify behaviors. [11] Thus, while following an algorithm may not be the main determinant of success, it is vital for residents to be able to plan accordingly and be adequately prepared should difficulties arise. Other work has demonstrated that residents showed an improvement in Anesthesia Non-Technical Skills (ANTS) and Anesthesia Crisis Resource Management (ACRM) after simulation-based training focusing on these skills. [31, 32] Our study attempted to assess the current state of resident attitudes and behaviors in our institution in order to improve the curriculum regarding urgent intubations OOOR.

Perhaps the most striking finding from our study is the large variation in resident performance in the same clinical scenario. Studies have shown that residents tend to exhibit training induced cognitive bias, implying they will preferentially choose a technique on which they received formal instruction. [25] As there is currently no standard way that we teach OOOR airway management, our findings were not surprising. Our study also showed that residents who felt they were more familiar with the content of the DAA, did not actually perform better than those who felt they were less familiar. It was also interesting that about one third of residents who did not follow the DAA appropriately did not alter their familiarity on the post-survey. This study reveals that even though this group of residents believed they were familiar with the content of the DAA, in the absence of any formal training in how to apply it, they did not follow it accurately in a simulated emergency setting.

Review of traditional anesthesiology training supports the theory that experience does not necessarily translate into expertise. [11] A study of anesthesiology residents in Denmark revealed that 97% of those tested could not recall the ASA DAA. [33] This study also showed that these same residents lacked the practical skills to handle a difficult airway during a simulated scenario. While some residents in this study did exhibit appropriate crisis resource management skills, this did not seem to correlate with sufficient knowledge of guidelines and practical skill. A review of non-technical skills by Flin et al. suggests that training of anesthesiology residents should be multifactorial, encompassing knowledge of guidelines, training in practical skills and education in crisis resource management and non-technical skills. [34] Residents in our study seemed to misjudge their familiarity and adherence to common guidelines and perhaps this is where anesthesiology resident training needs to shift its focus.

Similar to many other anesthesiology training programs in the United States, our institution does not have a dedicated curriculum to train residents in OOOR airway management, or in advanced airway management. Residents spend one month on a head and neck rotation during the CA1 year. There is a difficult airway course offered annually, but it is not mandatory for residents to attend. We saw in this study that attending this course did not impact whether or not a resident followed the DAA. This course does not provide instruction on how to approach intubations outside of the operating room. Residents learn these skills from experience and bedside instruction from senior residents and faculty. Programs that offer a curriculum in advanced airway management with defined objectives suggest that such training is crucial to increase airway management expertise. [30, 35] Such a curriculum should also consider the addition of other available difficult airway algorithms that may be more appropriate during these events, such as the Difficult Airway Society algorithm. [36] A focused curriculum may also improve understanding of complications resulting in and from unplanned tracheal extubation as well as enhancing accuracy in identifying the cricothyroid membrane. [37, 38] It will also be important to re-evaluate the curriculum in order to include new evidence, such as revisions to airway algorithms. [39] During such training, residents should also be instructed on the benefits of having faculty present and recognizing thresholds to involve faculty. [3, 20] Following traditional and simulation-based instruction, test-enhanced learning of critical action procedures may be used to increase retention. [40].

Another interesting finding was the similar success rates observed between junior and senior residents. This is relevant as only senior residents are permitted to intubate in the OOOR emergent setting at our institution, when perhaps there may be no difference in success rates and junior residents could benefit from the experience. Senior residents were found to be more likely to make an additional attempt with a different blade. This may reflect a higher level of comfort in the emergent setting and willingness to take the time to try an alternative technique.

There are several limitations to this study. The sample represents one group of residents at one institution and thus may not be generalizable to all practices. As not all residents were able to participate, this may introduce unknown biases if the missing residents were better or worse at OOOR than the residents who participated. There are also limitations inherent in any type of survey research. Report or social desirability bias could manifest in that residents may not want to report behaviors that place them in a potentially negative light. However, given that the surveys were completed anonymously, any influence of this bias is likely low. There is also limitation in correlating questionnaire responses with simulation performance. While residents were encouraged not to share details of the simulation scenario with their colleagues who had not yet completed the simulation., there may have been some discussion that occurred. Residents were divided into junior (CA1 and CA2) and senior (CA3) residents for purposes of analysis based on the policy at our institution that only CA3 residents are permitted to intubate during OOOR events. Resident exposure to the simulation environment is not standardized, and thus each resident’s prior experience with simulation may have affected their performance during the simulated scenario. The simulation scenario was standardized such that each resident had the identical set-up and progression of vital signs, and timing limits. The associated time limit may have prevented residents from performing actions they were planning to perform, and thus did not receive credit for them. Additionally, although evidence supports the use of simulation to identify critical gaps in performance and communication patterns, it is unclear how a resident’s performance in the simulator reflects their real-life performance for this specific scenario. [41, 42].

## Conclusions

To our knowledge, this appears to be the first study to evaluate anesthesiology resident performance of a simulated emergent airway outside of the operating room. While previous studies have shown improved compliance with DAAs following focused simulation curriculum and training, these studies have all looked at simulated cases within the operating room. Based on the results of this study, we have since developed a curriculum designed to improve resident comprehension of the difficult airway algorithm and its use OOOR, as well as enhance communication during stressful and often unorganized events. Following implementation of this innovative curriculum, we anticipate future studies that will compare questionnaire responses and simulation performances to examine efficacy of the new curriculum.

## Additional files

Additional file 1: Appendix 1. Pre-simulation survey. (DOCX 15 kb)

Additional file 2: Appendix 2. OOOR urgent intubation simulation scenario details. (DOCX 19 kb)

Additional file 3: Appendix 3. OOOR urgent intubation simulation checklist. (DOCX 18 kb)

Additional file 4: Appendix 4. Post-simulation survey. (DOCX 13 kb)
