# Supplementary material for: Assessing anesthesiology residents’ out-of-the-operating-room (OOOR) emergent airway management
Source: BMC Anesthesiol. 2017 Jul 15;17:96. doi: 10.1186/s12871-017-0387-2 (PMC5512836; doi:10.1186/s12871-017-0387-2)
Supplement: Supplementary file 1 — Appendix 1. Pre-simulation survey. (DOCX 15 kb) [file 12871_2017_387_MOESM1_ESM.docx]

Additional file 1: Appendix 1. Pre-simulation survey

Please answer these questions to the best of your ability.  Your responses from this survey will be linked with your performance during the simulator portion of this study.  This research is IRB exempt.

Doctor #:

In your experience, what is the most common indication for urgent intubation outside of the OR other than cardiac arrest?

- Hemodynamic instability
- Inability to protect airway
- Respiratory failure
- Unplanned extubation
- Other ____________________

Rank order your concerns when called to outside of the OR urgent intubations

______ Indication for intubation

______ Co-morbidities

______ Airway exam/history

______ Hemodynamics

______ Availability of assistance

______ Urgency of intubation

______ Other

From the options listed, what piece of patient information are you MOST interested in when called to intubate a patient outside of the OR?

- Indication for intubation
- Airway history
- Hemodynamic status
- Labs
- Other ____________________

How often do you perform a comprehensive airway exam on patients requiring urgent intubation outside of the OR?

- Never
- Rarely
- Sometimes
- Most of the Time
- Always

How often to you evaluate patients requiring urgent intubation outside of the OR for predictors of difficult mask ventilation?

- Never
- Rarely
- Sometimes
- Most of the Time
- Always

How often do you evaluate patients requiring urgent intubation outside of the OR for predictors of difficult cricothyroidotomy?

- Never
- Rarely
- Sometimes
- Most of the Time
- Always

How familiar are you with the ASA Difficult Airway Algorithm?

- Very unfamiliar
- Unfamiliar
- Neutral
- Familiar
- Very familiar

How accurately do you adhere to the ASA Difficult Airway Algorithm when performing outside of the OR urgent intubations?

- Never
- Rarely
- Sometimes
- Most of the Time
- Always

Are you aware of a Difficult Airway Algorithm specifically for trauma conditions?

- Yes
- No

Rank order your reasons for requesting faculty presence at outside of the OR urgent intubations.

______ History of difficult airway

______ Concern for difficult airway

______ Team conflict resolution

______ Additional set of hands

______ Patient instability

______ Other

How often do you request faculty presence at outside of the OR urgent intubations?

- Never
- Rarely
- Sometimes
- Quite Often
- Very Often

Rank order your reasons for requesting ENT presence at outside of the OR urgent intubations?

______ History of a diffiult airway

______ Concern for difficult airway

______ Potential for cricothroidotomy

______ Additional set of hands

______ Other

How often do you request ENT presence at outside of the OR urgent intubations?

- Never
- Rarely
- Sometimes
- Quite Often
- Very Often

How confident are you in your airway management skills during outside of the OR urgent intubations?

- No confidence
- Not very confident
- Neutral
- Pretty confident
- Very confident

How many outside of the OR urgent intubations have you participated in during your most recent 3 months at the Main?

- <5
- 5-10
- 11-15
- >15

Please complete the following demographic questions.

What is your current year of training?

- CA 1
- CA 2
- CA 3

Prior to your anesthesia residency, did you have exposure to airway management?

- Yes
- No

If you reported prior exposure to airway management, please select all that describe your role or involvement with prior airway management.

- EMT
- Respiratory therapist
- RN
- Other ____________________

Please complete the following questions regarding airway management instruction.

A lecture-based curriculum for airway management outside of the OR would be beneficial.

- Strongly Disagree
- Disagree
- Neither Agree nor Disagree
- Agree
- Strongly Agree

A simulation-based curriculum for airway management outside of the OR would be beneficial.

- Strongly Disagree
- Disagree
- Neither Agree nor Disagree
- Agree
- Strongly Agree

Have you attended the Difficult Airway Course sponsored by the Anesthesiology Department?

- Yes
- No

Did you receive any instruction regarding airway management for outside of the OR urgent intubations?

- Yes
- No

If you have received prior instruction, please describe from whom or in what setting you received instruction. Mark all that apply.

- Faculty
- Senior resident
- Lecture
- Simulation
- Independent reading
- Other ____________________
