# Supplementary material for: Assessing anesthesiology residents’ out-of-the-operating-room (OOOR) emergent airway management
Source: BMC Anesthesiol. 2017 Jul 15;17:96. doi: 10.1186/s12871-017-0387-2 (PMC5512836; doi:10.1186/s12871-017-0387-2)
Supplement: Supplementary file 2 — Appendix 2. OOOR urgent intubation simulation scenario details. (DOCX 19 kb) [file 12871_2017_387_MOESM2_ESM.docx]

Additional file 2: Appendix 2. OOOR urgent intubation simulation scenario details

**Pre-brief to resident**

You have just dropped a patient off in the BICU. You have finished handoff on that patient and are getting ready to go back to the OR. You are then asked by the RT/RN to evaluate another patient in the unit who is acutely desaturating. The anesthesia airway management team was called, they are currently at another code.

**Patient Details**

History

25yo M s/p motorcycle accident admitted today as a level 1 trauma. Skidded into a tree. Conscious at the scene when EMS arrived. No LOC. GCS 15. Hemodynamically stable.

Transferred to ER. Trauma work-up reveals:

No significant PMH or surgical history

NKDA

No home meds

Injuries: R tib/fib fracture, awaiting OR

C-spine not cleared due to distracting injury and neck tenderness.

No previous anesthetic history or airway history; missing some bottom teeth

Current vital signs

Afebrile HR 130, sinus BP 100/60 Spo2 85% on 10L FM

Labs

Labs – Hct 39%; K – 4.2; Cr – 0.9

ABG- 7.32/47/50 on 10L FM

Lines

One PIV

Arterial line

C-collar

Current status

Began to acutely desaturate, more obtunded, concern for aspiration and inability to protect his airway.

**Scenario programming**

| **Phase** | **Time** | **BP, HR, SpO2** | **Events** | **Learner actions** |
| --- | --- | --- | --- | --- |
| Baseline | 0-30 seconds | 100/60, 130, 85% | Time 0= learner enters bay  No change in vitals  Patient moans, not answering questions, moving extremities | Airway evaluation  Reviews history |
| 1 | 30-60 seconds | 100/60, 130, 75% | SpO2 decreases to 75% over 15 seconds  BP and HR remain unchanged  Patient moans, not answering questions, moving extremities | Begins patient and equipment preparation for intubation |
| 2 | 60-120 seconds | 100/60, 130, 70% | SpO2 decreases to 70% over 15 seconds BP and HR remain unchanged  Patient moans, not answering questions, moving extremities | Continues patient and equipment preparation for intubation |
| 3 | 120-240 seconds | 80/40, 100, 60% | BP, HR, SpO2 decreases to 60% over 15 seconds  Patient no longer moaning or moving | Airway management  Verification |
| 4 | 240-300 seconds | 70/30, 60, 60% | BP, HR, SpO2 decreases to 60% over 15 seconds  Patient no longer moaning or moving | Airway management  Verification  Terminate at 5-6 minutes if cricothyroidotmy not initiated – help will arrive |

**Sim Man Set-up**

Patient bed, sheets

C-collar

BP cuff

EKG leads

Facemask at 10L

PIV connected to Alaris pump

Arterial line

ACE wrap on R lower leg

**Supplies**

BP cuff, EKG, SpO2

Oxygen Face mask

PIV connected to Alaris

Arterial line

Ambu-bag with mask

Oral airway

Endotracheal tube(s)

Bougie

LMA

Laryngoscope

Glidescope – available out of room

Melker Cricothyrotomy kit – on crash cart

Suction with Yankauer

Capnogram

Crash cart – available out of room

Drugs from anesthesia cart

Airway equipment inside a tackle box or clear bin
