# Supplementary material for: Assessing anesthesiology residents’ out-of-the-operating-room (OOOR) emergent airway management
Source: BMC Anesthesiol. 2017 Jul 15;17:96. doi: 10.1186/s12871-017-0387-2 (PMC5512836; doi:10.1186/s12871-017-0387-2)
Supplement: Supplementary file 3 — Appendix 3. OOOR urgent intubation simulation checklist. (DOCX 18 kb) [file 12871_2017_387_MOESM3_ESM.docx]

Additional file 3: Appendix 3. OOOR urgent intubation simulation checklist

Doctor #

Year of anesthesia residency

- CA-1
- CA-2
- CA-3

Date of simulation testing:

Evaluator:

Airway Evaluation

|  | Yes | No |
| --- | --- | --- |
| Assesses mouth opening |  |  |
| Assesses mallampati score |  |  |
| Assesses dentition |  |  |
| Assesses T-M distance |  |  |
| Assesses neck extension |  |  |
| Notes C-collar |  |  |
| Inquires regarding prior difficult intubation |  |  |
| Inquires regarding prior difficult mask ventilation |  |  |
| Assesses NPO status |  |  |
| Listens to breath sounds |  |  |

Patient preparation

|  | Yes | No |
| --- | --- | --- |
| Moves bed away from wall |  |  |
| Removes headboard |  |  |
| Puts side rails down |  |  |
| Optimizes patient position |  |  |
| Supports patient's head/neck |  |  |

Equipment preparation

|  | Yes (1) | No (2) |
| --- | --- | --- |
| Suction catheter |  |  |
| Ambu-bag and mask |  |  |
| Oral airway |  |  |
| ETCO2 detector |  |  |
| Laryngoscope |  |  |
| ETT |  |  |
| Reviews vital signs |  |  |
| IV access |  |  |
| Advanced airway equipment |  |  |
| Prepares medications |  |  |
| Requests crash cart |  |  |

Airway management

|  | Yes | No | N/A |
| --- | --- | --- | --- |
| Discusses plan with team |  |  |  |
| Pre-oxygenates |  |  |  |
| Attempts BMV |  |  |  |
| First attempt DL |  |  |  |
| First attempt glidescope |  |  |  |
| Second attempt with different blade |  |  |  |
| Successful intubation |  |  |  |
| Listens to breath sounds |  |  |  |
| Requests faculty presence |  |  |  |
| Requests ENT presence |  |  |  |
| Considers/places LMA |  |  |  |
| Considers/places alternate device |  |  |  |
| Considers/initiates cricothyroidotomy |  |  |  |
| Discusses subsequent plan with team after initial attempts unsuccessful |  |  |  |
