# Supplementary material for: Assessing anesthesiology residents’ out-of-the-operating-room (OOOR) emergent airway management
Source: BMC Anesthesiol. 2017 Jul 15;17:96. doi: 10.1186/s12871-017-0387-2 (PMC5512836; doi:10.1186/s12871-017-0387-2)
Supplement: Supplementary file 4 — Appendix 4. Post-simulation survey. (DOCX 13 kb) [file 12871_2017_387_MOESM4_ESM.docx]

Additional file 4: Appendix 4. Post-simulation survey

Based upon your experience during this simulated outside the OR urgent intubation, please answer the following questions.  Your responses will be correlated with your performance and you previous survey answers and will be used to enhance our airway management training curriculum.

Doctor #:

The simulated scenario was realistic.

- Strongly Disagree
- Disagree
- Neither Agree nor Disagree
- Agree
- Strongly Agree

How familiar are you with the ASA Difficult Airway Algorithm?

- Very unfamiliar
- Unfamiliar
- Neutral
- Familiar
- Very familiar

I used the ASA Difficult Airway Algorithm during the simulated scenario.

- Yes
- No

If you used the ASA DAA during the scenario, how accurately did you follow the algorithm?

- Never
- Rarely
- Sometimes
- Most of the Time
- Always

I have encountered a cannot intubate/cannot ventilate situation previously (either in the OR or outside the OR)

- Yes
- No

I have practiced cricothyroidotomy techniques in the simulation center before.

- Yes
- No

I have performed a cricothyroidotomy on a patient before.

- Yes
- No
